# Minocycline Protects against Rotenone-Induced Neurotoxicity Correlating with Upregulation of Nurr1 in a Parkinson's Disease Rat Model

**DOI:** 10.1155/2019/6843265

**Published:** 2019-03-05

**Authors:** Congcong Sun, Yun Wang, Mingshu Mo, Chengyuan Song, Xingbang Wang, Si Chen, Yiming Liu

**Affiliations:** ^1^Department of Neurology, Qilu Hospital of Shandong University, Jinan 250012, Shandong, China; ^2^Jiangsu Key Laboratory of New Drug Research and Clinical Pharmacy, Xuzhou Medical University, Xuzhou 221002, Jiangsu, China; ^3^Department of Neurology, First Affiliated Hospital of Guangzhou Medical University, Guangzhou 510120, Guangdong, China

## Abstract

The aim of this study was to investigate the effect of minocycline in rats with rotenone-induced Parkinson's disease (PD). The open field test was performed to determine the motor ability of the rats. Double immunofluorescence staining was used to detect the expression of tyrosine hydroxylase (TH) and Nurr1 in the substantia nigra (SN) of rats. The relative protein levels of TH, Nurr1, and the total- and phosphorylated-cAMP-response element binding protein (CREB) were determined by western blot analysis. The production of reactive oxygen species (ROS) and nitric oxide (NO) was detected by commercial kits. After exposure to rotenone for 28 days, rats exhibited decreased ambulation and rearing frequency and prolonged immobility time with loss of TH positive neurons in the SN. The phosphorylation levels of CREB and Nurr1 expression decreased significantly accompanied with the release of ROS and NO. Minocycline alleviated the motor deficits of rats lesioned by rotenone and elevated the expression of TH, as well as suppressing the release of ROS and NO in the SN. That was in line with the elevated phosphorylation levels of CREB and Nurr1 expression. In conclusion, our present study showed minocycline protected against neurotoxicity in a rotenone-induced rat model of PD, which was correlated with upregulation of Nurr1.

## 1. Introduction

Parkinson's disease (PD) is a chronic progressive neurodegenerative disease featured with degeneration of dopaminergic (DA) neurons in the substantia nigra (SN) [1]. After degeneration of more than 80% of the DA neurons, the clinical motor symptoms, such as bradykinesia, muscular rigidity, rest tremor, and postural and gait impairment, become apparent [2]. Levodopa is still the primary treatment for PD [3]. Enhancing neuroprotection at the early pathogenic stage is crucial for delaying the development of PD.

Although the pathological mechanisms of PD are not well understood, it is widely accepted that oxidative stress plays important roles in the initial degeneration of DA neurons [4]. Oxidative stress is remarkably increased in the brain tissue of patients with PD [5]. Excessive production of reactive oxygen species (ROS) and nitric oxide (NO) triggers cellular damage through lipid peroxidation, protein oxidation or nitration, mitochondrial dysfunction, and DNA fragmentation [6, 7].

The transcription factor Nurr1 expressed in the DA neurons regulates resistance to oxidative stress and the expression of TH, a rate limiting enzyme catalyzing neuronal dopamine synthesis [8, 9]. Mutations in Nurr1 are associated with the pathogenesis of familial PD cases [10]. Nurr1 deficiency may lead to impaired DA release, before significant loss of DA neurons [11]. Nurr1 agonists improve the behavioral deficits in animal models [12]. Therefore, Nurr1 is a feasible and effective drug target for neuroprotection in PD.

Minocycline is a semisynthetic tetracycline with high lipophilicity [13]. In addition to its antimicrobial activity, it presents a neuroprotective capacity [14]. Several studies reported that minocycline increased the phosphorylation of the cAMP-response element binding protein (CREB) in rodents subjected to ischemia, *β*-amyloid toxicity, and prion infection [15–17]. Phosphorylated-CREB could promote the Nurr1 expression via binding to its promoters [18]. Whether minocycline influences the expression of these proteins in degenerating DA neurons remains elusive. Rotenone is an environmental neurotoxin, which causes oxidative stress and is widely used in modeling the pathogenesis of PD [19]. Here, we investigated the effect of minocycline in rotenone-induced rat models of Parkinson's disease.

## 2. Materials and Methods

### 2.1. Animals and Drugs

Male Wistar rats (weighing 270-290 g) were procured from the Experimental Animal Center of Shandong University (Jinan, Shandong, China). Animal experiments and procedures were approved by the Animal Experimentation Ethics Committee of Shandong University. The rats were allowed free access to food and water ad libitum, under standard temperature and humidity, with the 12 h light/dark cycle.

Minocycline (Sigma-Aldrich, St. Louis, MO, USA) was dissolved in a 0.9% saline solution (10 mg/ml). Rotenone (Sigma-Aldrich) was dissolved in sunflower oil (2 mg/ml).

### 2.2. Experimental Design

Rats were randomly divided into four groups labeled as control (no treatment, n=20), minocycline (daily intraperitoneal injection of minocycline, 30 mg/kg, n=20), rotenone (daily subcutaneous administration of rotenone, 1.5 mg/kg, n=40), and minocycline plus rotenone (intraperitoneal injection of minocycline one day before rotenone administration, 30 mg/kg and 1.5 mg/kg, respectively, n=40). At the 28th day of the experiment, the open field test was performed to assess spontaneous locomotor activity.

### 2.3. Open Field Test

A square apparatus (80 cm × 80 cm×40 cm) was divided into 16 equal squares. The rat was placed in the central area of the open field and allowed to explore the area freely. Ambulation (the number of the square that the rat crossed), the number of rearing, and immobility time were recorded for 3 min. The apparatus was washed after testing each rat to eliminate the possible odor left.

### 2.4. Immunofluorescent Staining and Analysis

After anesthetizing with 10% chloral hydrate (5 ml/kg, intraperitoneal injection), rats were sacrificed and perfused with saline followed by 4% paraformaldehyde through the left ventricle of the heart. Brains were obtained and fixed in 4% paraformaldehyde overnight and then kept in a 0.1 M PBS-sucrose solution (20% and 30%) at 4°C until they were immersed. Samples were embedded in optimal cutting temperature compound and frozen sections (10 *μ*m) were prepared with a Cryostat Microtome (CM1900, Leica, Solms, Germany) according to the standard procedures. After incubation with 0.3% Triton X-100 (v/v), sections were blocked with 10% normal goat serum (Kirkegaard & Perry Laboratories, Inc., Maryland, WA, USA) and then incubated with rabbit anti-rat anti-TH (1:500 dilution; Abcam, Cambridge, MA, USA) and mouse anti-rat anti-Nurr1 (1:300 dilution; Abcam) primary antibodies overnight. After washing with 0.01 M PBS, sections were incubated with FITC-conjugated goat anti-rabbit secondary antibody and Alexa Fluor 594-conjugated goat anti-mouse secondary antibody (1:1,000 dilution; Jackson ImmunoResearch Inc., West Grove, PA, USA). After washing with 0.01 M PBS, the sections were counterstained with 4′,6-diamidino-2-phenylindole dihydrochloride (DAPI, 1:10,000 dilution; Sigma-Aldrich) and then examined under a microscope (BX51, Olympus, Tokyo, Japan). The number of immunostaining positive cells was counted by the Image Pro Plus image analysis software (Media Cybernetics, Silver Spring, MD, USA) in comparable fields (300x210 um, 3 fields per section x 3 sections per rat) and was presented as the average cell number per field on each section.

### 2.5. Western Blot Analysis

The SN tissues were rapidly dissected from the brain and homogenized with a lysis buffer containing a protease inhibitor cocktail (Roche, Basel, Switzerland). The protein concentration of the supernatant was determined using the Bicinchoninic Acid (BCA) Protein Assay Kit (Thermo Fisher Scientific, Rockford, IL, USA). A total of 50 *μ*g protein was separated by 10%-15% SDS-polyacrylamide gels and then transferred onto the polyvinylidene difluoride (PVDF) membrane (Millipore, Bedford, MA, USA). The membrane was incubated with the following primary antibodies: rabbit anti-rat anti-TH (1:1,000 dilution; Abcam), mouse anti-rat anti-Nurr1 (1:1,000 dilution; Abcam), rabbit anti-rat anti-CREB (1:1,000 dilution; Cell Signaling Technology, Danvers, MA, USA), rabbit anti-rat anti-phospho-CREB (Ser133) (1:1,000 dilution; Cell Signaling Technology), and mouse anti-rat anti-*β*-actin (1:10,000 dilution; Proteintech Group Inc.). Subsequently, the membrane was washed and incubated with horseradish peroxidase- (HRP-) conjugated anti-mouse IgG or anti-rabbit IgG (KPL, Gaithersburg, MD, USA) secondary antibodies. Protein bands were detected by the SuperSignal® West Pico Chemiluminescent Substrate (Thermo Fisher Scientific Inc.). The band densities were calculated as the expression ratios of the target protein to *β*-actin using the ImageJ 1.42q software (US National Institutes of Health).

### 2.6. Measurement of ROS and NO Levels in the SN of Rats

ROS and NO production were measured using a ROS assay kit and NO assay kit (nitrate reductase method) (Nanjing Jiancheng Bioengineering Institute, China), respectively. Briefly, the single cell suspension was prepared from the fresh SN tissue by the mechanical trituration method. The protein concentration of the supernatant was determined using the BCA Protein Assay Kit. 10 uM DCFH-DA was added to the suspended cells and incubated for 45 min at 37°C. 5 uM DAF-FM DA was incubated with the suspended cells for 20 min at 37°C. After washing with PBS, the fluorescence intensity was measured by a microplate reader (at 485 nm excitation and 535 nm emission for ROS and at 495 nm excitation and 515 nm emission for NO). The levels of ROS and NO were expressed as fluorescence intensity/mg protein and umol/g protein, respectively.

### 2.7. Statistical Analysis

Data are expressed as mean ± standard error of the mean (SEM). Statistical analysis was performed with the SPSS 12.0 software (SPSS Inc., Chicago, IL, USA). One-way analysis of variance (ANOVA) was performed for group comparison, followed by the Tukey test or the Games-Howell test for post hoc comparison.* p*<0.05 was considered as statistically significant.

## 3. Results

### 3.1. Minocycline Alleviated Behavioral Deficits in Rats following Rotenone Treatment

**Figure 1:**
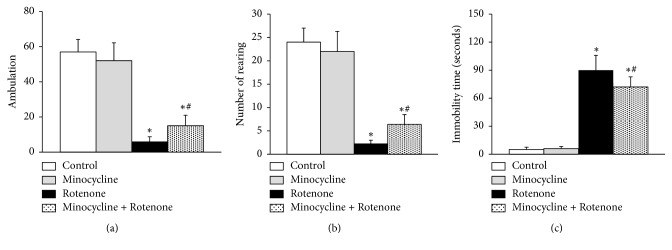
Minocycline alleviated motor deficits of rats treated with rotenone. The open field test results showing the number of ambulation and rearing and the immobility time of rats (n=15 for each group) within 3 min. ^*∗*^*p*<0.05, compared to the control group; ^#^*p*< 0.05, compared to the rotenone-alone-treated group.

The open field test was performed to evaluate the spontaneous motor ability of the rats (15 rats in each group). Compared to the control, rats exposed to rotenone showed an obvious reduction in the number of ambulation and rearing and prolonged periods of immobility (^*∗*^*p*<0.05, Figure 1). Treatment with minocycline before rotenone administration increased the number of ambulation and rearing of rats and shortened the immobility time (#*p*<0.05, Figure 1). No obvious differences were observed between the control group and the minocycline group in the above indexes. These results indicated that minocycline might, to some extent, enhance the motor ability of PD rat models.

### 3.2. Minocycline Alleviated Oxidative Stress

**Figure 2:**
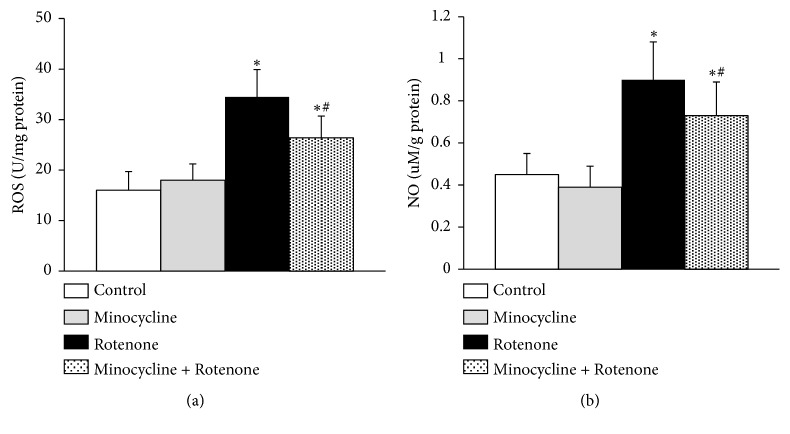
The production of reactive oxygen species (ROS) and nitric oxide (NO) in the SN of rats. The production of ROS (a) and NO (b) in the SN of rats (n=5 for each group) measured with a commercial ROS assay kit and NO assay kit. ^*∗*^*p*<0.05, compared to the control group; ^#^*p*<0.05, compared to the rotenone-alone group.

ROS and NO assay kits were used to determine the production of ROS and NO in the SN of rats (n=5 for each group). Our results showed that rotenone led to a significant increase in ROS and NO levels compared to control (^*∗*^*p*<0.05, Figure 2) and minocycline treatment before rotenone administration reversed the increase significantly (^#^*p*<0.05, Figure 2). No obvious differences in ROS or NO levels were observed between the control group and the minocycline group. These findings indicated that minocycline inhibited the production of ROS and NO in the SN of rats treated with rotenone.

### 3.3. Minocycline Increased the Expression of TH and Nurr1

**Figure 3:**
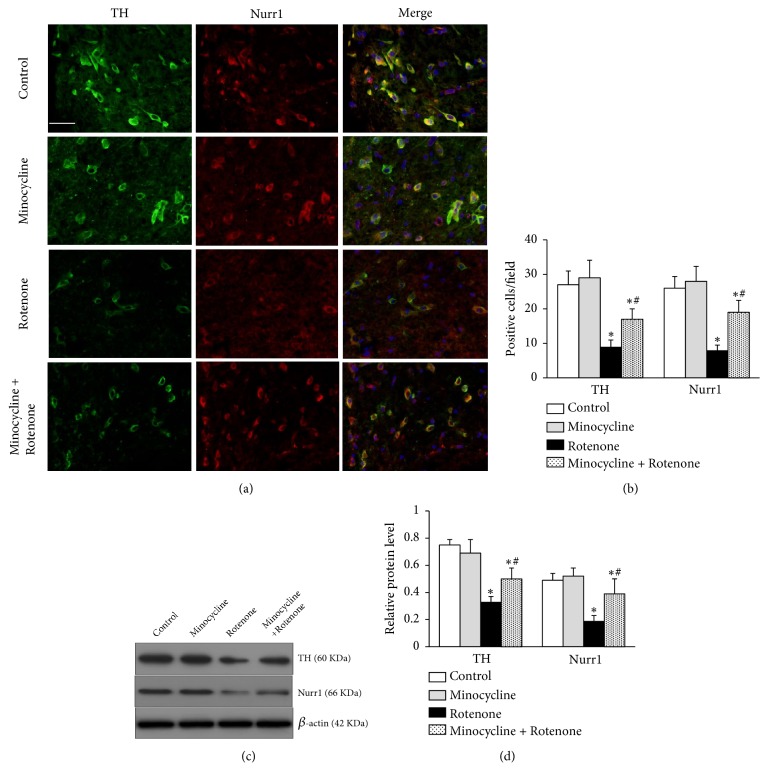
Expression of tyrosine hydroxylase (TH) and Nurr1 in the SN of rats. (a) Double immunostaining for TH and Nurr1 in the SN of rats (×400; green, TH; red, Nurr1; blue, staining with DAPI; scale bar = 50 um). (b) The number of immunostaining positive cells for TH and Nurr1 in the SN of rats (n=5 for each group). (c) The protein expression of TH and Nurr1 in the SN of rats determined by western blot analysis. (d) The relative band intensities of TH and Nurr1 normalized to the expression of *β*-actin (n=5 for each group). ^*∗*^*p*<0.05, compared to the control group; ^#^*p*<0.05, compared to the rotenone-alone-treated group.

The expression of TH and Nurr1 in the SN of rats was detected by immunofluorescent staining and western blot analysis. Our results showed that Nurr1 was expressed in TH positive neurons. After treatment with rotenone, obvious loss of TH positive neurons and the diminished expression of Nurr1 were observed in the SN of rats (n=5 for each group, ^*∗*^*p*<0.05, Figures 3(a) and 3(b)), which were alleviated by minocycline treatment before rotenone administration (n=5 for each group, ^#^*p*<0.05, Figures 3(a) and 3(b)). Compared to the control group, the relative protein levels of TH and Nurr1 were significantly decreased in rats exposed to rotenone (n=5 for each group, ^*∗*^*p*<0.05, Figures 3(c) and 3(d)). Treatment with minocycline significantly increased TH and Nurr1 expression in the SN compared to rats exposed to rotenone alone (n=5 for each group, ^#^*p*<0.05, Figures 3(c) and 3(d)). No obvious differences in the TH and Nurr1 expression were observed between the control group and the minocycline group (n=5 for each group,* p*>0.05, Figure 3). These results indicated that minocycline treatment increased the TH and Nurr1 expression in the SN of rats exposed to rotenone.

### 3.4. Minocycline Upregulated the Phosphorylation Level of CREB

**Figure 4:**
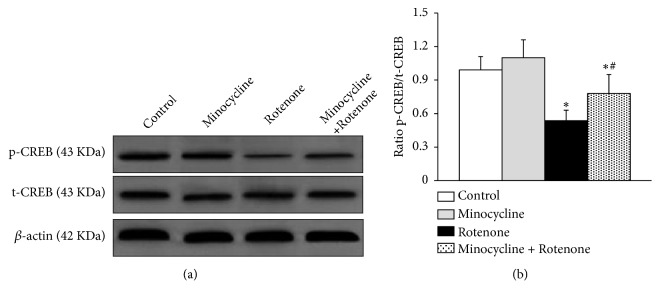
Expression of the total-cAMP-response element binding protein (t-CREB) and phosphorylated-cAMP-response element binding protein (p-CREB) in the SN of rats. (a) The protein expression of t-CREB and p-CREB in the SN of rats (n=5 for each group) determined by western blot analysis. (b) Ratio of p-CREB/t-CREB in the SN of rats. Significantly decreased p-CREB/t-CREB ratio was observed in the SN of rats exposed to rotenone compared to the control group, whereas it was higher in the minocycline-plus-rotenone group compared to the rotenone group. ^*∗*^*p*<0.05, compared to the control group; ^#^*p*<0.05, compared to the rotenone-alone group.

The expression of total-CREB (t-CREB) and phosphorylated-CREB (p-CREB) in the SN of rats was detected by western blot analysis. Neither rotenone nor minocycline produced obvious effect on the expression of total-CREB. Compared to the control group, the p-CREB/t-CREB ratio was significantly reduced in rats treated with rotenone (n=5 for each group, ^*∗*^*p*<0.05, Figure 4), and this effect was abolished by minocycline treatment (n=5, ^#^*p*<0.05, Figure 4). That indicated minocycline upregulated the phosphorylation level of CREB.

## 4. Discussion

The present study indicated that minocycline protected against rotenone-induced neurotoxicity in a rat model of PD. It alleviated the motor deficits of rats and increased the expression of TH and suppressed the oxidative stress in the SN. That was consistent with the upregulated phosphorylation levels of CREB and Nurr1 expression. TH positive neurons highly expressing Nurr1 were abundant in the SN of normal rats. Nurr1 is essential for the development and maintenance of DA neurons [20]. Interestingly, it also protects the DA neurons by decreasing the production of intracellular ROS [21]. NO is associated with increased dopaminergic damage in Nurr1 heterozygous mice [22]. Similar to other studies [23], we found Nurr1 expression was remarkably decreased in the SN of rats after exposure to rotenone. This finding was in line with the loss of TH positive neurons and the increased oxidative stress. Many studies have reported that minocycline protects the DA neurons from neurotoxins [24–26]. In the present study, we found minocycline treatment before rotenone administration increased the expression of Nurr1. This was accompanied with decreased content of ROS and NO and increased TH positive cells. These results implied that minocycline protected DA neurons against rotenone neurotoxicity, which was correlated with upregulation of Nurr1.

CREB plays pleiotropic roles in the nervous system [27]. ROS and NO modulate many signaling targets in the nervous system, including increasing CREB phosphorylation [28, 29]. In turn, CREB functions as a pivotal upstream integrator of neuroprotective signaling against oxidative stress-mediated neuronal cell death [30]. Redundant ROS and NO clearly result in cellular damage, although other feedback mechanisms might alleviate their toxic effects. In our study, decreased CREB phosphorylation was detected in the SN of rats treated with rotenone, which is similar to the effects of 6-OHDA and MPTP [31, 32]. We also found that minocycline treatment before rotenone administration increased the ratio of p-CREB/t-CREB. This result is consistent with the in vivo studies performed under different pathological states [15–17]. The Nurr1 gene possesses CAMP-response element sequences that bind p-CREB to promote its expression [18]. That may partially explain the effect of minocycline in upregulating the Nurr1 and TH expression.

Although many studies have reported that minocycline plays neuroprotective roles, it has not been shown to improve the motor function in patients with early PD in the clinic [33]. Since most DA neurons would have been degenerated when patients present motor symptoms, drug delivery at this stage might be too late. Our results showed that minocycline early treatment improved the motor deficits of rats suffering from rotenone toxicity, although the improvement was not satisfactory. Whether minocycline serves as an auxiliary treatment deserves further exploration.

## 5. Conclusions

This study showed minocycline could protect against rotenone-induced neurotoxicity in a rat model of Parkinson's disease. It alleviated motor deficits and elevated the TH expression, as well as suppressing the oxidative stress. That was correlated with upregulation of Nurr1. Whether minocycline is useful as adjuvant therapy for Parkinson's disease deserves further exploration.

## Data Availability

The data used to support the findings of this study are available from the corresponding author upon request.
